# BioSig: The Free and Open Source Software Library for Biomedical Signal Processing

**DOI:** 10.1155/2011/935364

**Published:** 2011-03-08

**Authors:** Carmen Vidaurre, Tilmann H. Sander, Alois Schlögl

**Affiliations:** ^1^Machine Learning Group, Berlin Institute of Technology, Department of Software Engineering and Theoritical Computer Science, 10587 Berlin, Germany; ^2^Physikalisch-Technische Bundesanstalt Institut Berlin, 10587 Berlin, Germany; ^3^Institute of Human Computer Interfaces, Graz University of Technology, Krenngasse 37, 8010 Graz, Austria; ^4^Institute of Science and Technology Austria (IST Austria), Am Campus 1, 3400 Klosterneuburg, Austria

## Abstract

BioSig is an open source software library for biomedical signal processing. The aim of the BioSig project is to foster research in biomedical signal processing by providing free and open source software tools for many different application areas. Some of the areas where BioSig can be employed are neuroinformatics, brain-computer interfaces, neurophysiology, psychology, cardiovascular systems, and sleep research. Moreover, the analysis of biosignals such as the electroencephalogram (EEG), electrocorticogram (ECoG), electrocardiogram (ECG), electrooculogram (EOG), electromyogram (EMG), or respiration signals is a very relevant element of the BioSig project. Specifically, BioSig provides solutions for data acquisition, artifact processing, quality control, feature extraction, classification, modeling, and data visualization, to name a few. In this paper, we highlight several methods to help students and researchers to work more efficiently with biomedical signals.

## 1. Introduction

The area of biomedical signal processing has to deal with a large variety of topics. Artifact contamination, low signal-to-noise ratios, different data formats, classification, and statistical evaluation are general challenges of the field. Furthermore, a large number of different data processing methods for different signal modalities (EEG, ECG, etc.) and for different applications has to be considered. Moreover, software development itself is an important part of biomedical signal processing.

In the 1990s, the use of Matlab became popular to process biosignals. However, the algorithms were rarely available and the reimplementation of methods was common, even within the same research group. The field of software development was characterized by providers that offered closed (proprietary) solutions. This caused incompatibilities, and the same algorithms were implemented again and again. Another side effect was that each equipment provider defined its own data format for storing biosignals. These data could then be analysed only with the proprietary software of the vendor. Data export, if possible, was difficult and resulted usually in loss of information (e.g., metadata about the recording conditions, like filter settings or sampling rate, were not preserved).

These facts made the development and validation of new methods difficult. Additionally, the success of free and open source software in the field of operating systems (e.g., Linux) and server software did encourage the development of a free software library for biomedical signal processing.

Despite its focus on EEG data, BioSig can be used for general signal processing tasks related to a variety of measurement modalities. One example is the calculation of event-related averages in MEG. Another one is the calculation of spectral estimates of individual channels or time segments in functional near-infrared spectroscopy data. BioSig covers many EEG and polygraphic data formats. Furthermore, data loading is accomplished by a simple command.

BioSig consists of some (more or less) coherent parts, that are summarized as follows.

- *BioSig for Octave and Matlab (biosig4octmat)*. A toolbox for Octave and Matlab with powerful data import and export filters, feature extraction algorithms, classification methods, and a powerful viewing and scoring software.
- *BioSig for C/C++ (biosig4c++)*. A C/C++ library that provides reading and writing routines for different biosignal data formats.
- *rtsBCI (rtsbci)*. A real-time Brain Computer Interface (BCI) system implemented in Matlab and Simulink.

Most functions implemented in BioSig can be used with both Matlab and Octave and are installed through the package “biosig4octmat”. This is also the main module of the project. Within this library many data formats are supported, and the toolbox provides a common interface for reading [1] different formats. An automated detection of the file format eases the use, making the detection transparent to the user. The writing of several common file formats is also supported. Additionally, useful algorithms for artifact detection and correction are available. Many algorithms for stochastic model parameters (autoregressive, multivariate, time-varying, etc.) are accessible in the time series analysis (TSA) [2] toolbox. These and other functions from the NaN-toolbox [3] are able to handle data with missing values (caused by, e.g., artifacts), too.

BioSig software is available “on-line” (cf. [4]) and under the terms of the “General Public License” (GPL) v3 [5]. The GPL guarantees to the users that the BioSig library can be freely used, studied, modified, and distributed. Having a library for biomedical signal processing provides a summary of prior art in the field and might be helpful against the detrimental effects of software patents.

## 2. Structure of BioSig

### 2.1. Toolbox Components

 Matlab is a widespread numerical programming language used for biosignal processing, therefore BioSig started being developed for this proprietary platform. However, in order to provide a really free and open library, a special effort was undertaken to provide compatibility with Octave [6], a free and largely compatible alternative to Matlab. All functions are tested for their compatibility with both platforms. Although BioSig supports other programming languages such as C/C++ or Python, the main module of BioSig is for Matlab and Octave and we will focus on this in the following.

BioSig covers many aspects of biomedical signal processing. Therefore, the toolbox is divided into subcategories which depend on the functionally of the algorithms contained in them. After installing BioSig, the following folders are available and ordered by subtasks:

- file access, data input and output (loading and saving routines), path: biosig/t200/*,
- preprocessing, quality control, and artifact processing, path: biosig/t250/*,
- signal processing and feature extraction, path: biosig/t300/*,
- event-related synchronization/desynchronization (ERS/D) maps, path: biosig/t310/*,
- classification and statistics (single trial analysis), path: biosig/t400/*,
- statistical tests, path: biosig/t450/*,
- evaluation criteria, path: biosig/t490/*,
- visualization, path: biosig/t500/*,
- time series analysis, path: tsa/*,
- statistics of data with missing values encoded as NaN (not a number), path: nan/*,
- interactive viewer and scoring (requires Matlab), path: biosig/viewer/*,
- documentation and help, path: biosig/doc/*.

**Figure 1:**
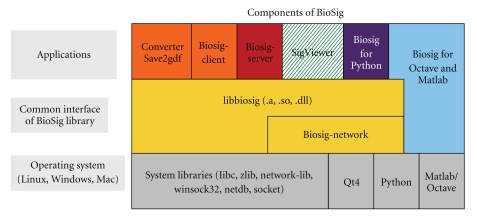
Architecture of the BioSig toolbox and its elements.

Figure 1 represents a scheme of the toolbox and how its different elements are interrelated. 

The module “data input and output” is a common interface for accessing the various formats including an automated format detection. It supports reading of about 40 and writing of 10 different data formats including some audio formats. The preprocessing module provides tools for triggering (segmenting) signal data, for artifact detection, artifact reduction and quality control. The signal processing module includes several specialized biosignal processing functions, but also interfaces to standard signal processing functions and wrapper functions for more complex analyses. The classification module includes support for different classification methods. A number of classifiers including linear, quadratic and regularized discriminant analysis, several methods of Support Vector Machines (SVM), Naive Bayes Classifiers, Perceptron Learning, Partial Least Squares/Regression analysis as well as some sparse classifiers are supported; cross-validation procedures are supported to prevent overfitting. More recently, these methods were extended for the use with missing values and are now also distributed as part of the NaN-toolbox [3]. The module on evaluation criteria contains several functions for performance metrics as used in the field of Brain-Computer Interface (BCI) research [7]. The visualization module contains a simple viewer for biomedical data as well as a wrapper function to visualize the results of several standard analysis procedures. The interactive viewing and scoring software (SViewer) is based on the graphical user interface of Matlab, which is currently not supported by Octave. An alternative is the free stand-alone viewing and scoring software “SigViewer”.

Other important modules of BioSig are the Time Series Analysis (TSA) toolbox [2] and the NaN-toolbox [3], which are also part of the Octave-forge repository. The TSA toolbox provides a unique variety of coupling measures based on a multivariate autoregressive modeling routine. The NaN toolbox handles data with missing values, which are commonly caused by artifacts and encoded by not-a-number (NaN). BioSig contains also several demonstration examples and a benchmark function for comparing the performance of different platforms. The benchmark functions perform some typical processing steps for calculating the classifier of BCI experiment. First some data is loaded; then several features are extracted; the features are used to compute a classifier; a cross-validation procedure (in this case a leave-one-out method) is used for validating the classifiers. The benchmark can be used to compare different hardware platforms as well as different versions of Octave and Matlab.

Contemporary high-density EEG and MEG can result in very large multidimensional signal vectors. As a consequence their processing might require large amounts of memory only available within 64 bit operating systems. To facilitate work with simpler hardware, BioSig can read data in blocks and the user has to control that parameter extraction is consistent across blocks of data. Reading of individual channels is possible even if the full data set does not fit into memory. This is accomplished by consecutive reading of blocks and concatenation of single channel data slices.

### 2.2. Compatibility between Octave and Matlab

 Making BioSig compatible to Octave and Matlab was not self-evident. In the past, a number of core functions present in Matlab were missing from Octave, and had to be provided by BioSig for full compatibility. Luckily, the number of missing functions has been strongly reduced with newer versions of Octave (v3.2 and higher).

In addition, another problem with proprietary Matlab was addressed, notable not all Matlab users have all toolboxes available. Additional effort was spent to replace the dependency on add-on toolboxes (like statistics and signal processing) with free alternatives. This effort resulted in the release of “free toolboxes for Matlab” (freetb4matlab), which makes toolbox from Octave and Octave-forge available for the use with Matlab.

In general, the attempt to make BioSig (in particular biosig4octmat) fully compatible to Octave as well as Matlab was widely successful. Currently, only the interactive scoring software (a desirable but not mission-critical component) cannot be used with Octave. BioSig demonstrates that also a large-scale project can be programmed in such a way that it can run on Matlab as well as Octave without any code modifications.

### 2.3. Compatibility of BioSig with Other Toolboxes

Clearly the BioSig is one of several toolboxes designed for biomedical signal analysis. This suggests that interdependency between toolboxes and avoiding redundancy are quite important, but so far this topic has been rather neglected. A promising example is the reliance of FieldTrip (http://fieldtrip.fcdonders.nl/) and SPM (http://www.fil.ion.ucl.ac.uk/spm) on the BioSig for specific tasks. Examples are the reading of various file formats only supported in the BioSig (cf. FieldTrip: ft_read_data.m) and the multivariate autoregressive modeling implemented in the BioSig (cf. FieldTrip: ft_mvaranalysis.m). Additionally, Biosig is also included in EEGlab, a widely used interactive Matlab toolbox for EEG and MEG processing.

Dependencies like these need to be better addressed as no single toolbox provides all possible types of analysis. The range of processing steps appropriate for bioelectric and biomagnetic signals as summarized in [8] clearly exceeds the scope of a single toolbox. Unlike for proprietary software, Toolbox design is not “one against the others race”, but rather a cooperative effort to create “scratch an itch” of developers and users, and eventually build the “super tool” for everyone.

 A related question is the proper acknowledgment of a toolbox and its authors in the scientific literature. A toolbox typically implements several tens to at most a few hundred published algorithms. An interesting idea, is that the toolbox provides a “log of methods” used by an application to the user. Currently, BioSig cites the publications in the documentation of each function. In this way, the authors of the original works can be acknowledged. It would be desirable, that also the published software and its authors are properly cited.

## 3. Data Formats

**Figure 2:**
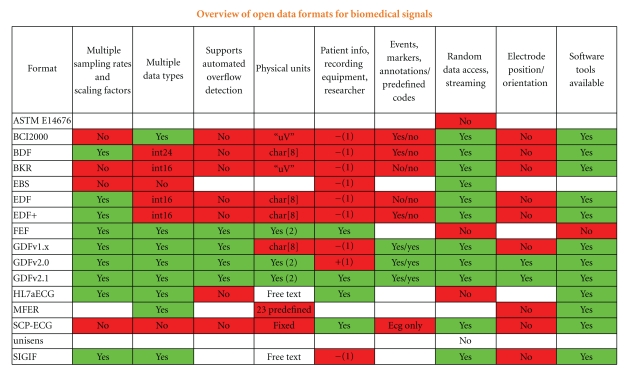
Properties of open and vendor independent data formats.

 Biomedical signals are stored in many different data formats. Most formats have been developed for a specific purpose of a specialized community (ECG research, EEG analysis, sleep research, etc.), by companies, research groups, and standardization organizations. A detailed comparison of about 20 biomedical data formats with publicly available specifications is shown in Figure 2 (for more details see [9]). 

Although BioSig supports over 40 different data formats and can ease the problem, still the definition of a general purpose format was needed. In order to overcome the proliferation of data formats a “General Data Format for biosignals” (GDF) [1] has been developed with the aim to combine the best features of different formats into a single data format. BioSig provides a common interface for different data formats including an automated identification of the file format. This provides a seamless user interface, specifically the user can utilize the same functions for reading different formats.

Version 1 of the General Data format (GDF), [10], has been developed and successfully implemented and used in BCI research. GDF provides many useful features (different sampling rates and calibration values for different channels, an automated overflow detection, support of different data types, encoding of filter settings, etc.), that are only partly implemented in other formats. A key idea is also to define a fixed coding scheme for events, which supports compatibility of event information across different studies and laboratories. GDF is the first data format that addresses this topic.

Within recent years, new requirements became apparent. The new Version 2 of the GDF addresses the need for:

- subject-specific information (gender, age, impairment, etc.),
- recording location, identification of recording software, and so forth,
- possibilities for storing the electrode positions in spatial coordinates, electrode impedances, and so forth,
- more efficient encoding of date and time, physical dimensions, and filter information,
- nonequidistant (sparse) sampling.

The structure of GDF v2.0 is similar to EDF [11], GDF1.x [10], and EDF+ [12].

Briefly, an GDF file consists of the following five components: the fixed header or header 1 (with 256 bytes) is mandatory, the variable header or header 2 containing channel-specific information (number-of-channels times 256 bytes), the tag-length-value (TLV) header or header 3 contains optional information, the data section, and the table of events. Header 2 can be empty, in case that no channel information is stored (e.g., in pure event files).

Data is stored in little endian format. However, BioSig supports also big-endian platforms by converting the data internally. The Version field is of type char [8] and is stored at the beginning of the file. This field is used to provide upwards compatibility with past and future versions of GDF.

The format definition of GDF is nearly as simple as the definition of EDF. The use of binary encodings enables a more compressed representation; accordingly, more information can be stored within the header information. This enables a higher accuracy (e.g., in date and time information) and additional information can be stored without extending the header size.

The proposed format specification was successfully implemented in C/C++ as well as an *M*-file which can be used with Octave (>2.9.12) and Matlab (>6.5). The software implementation requires only minor changes to upgrade from EDF, BDF, or earlier GDF to GDF 2.20. In BioSig different data formats can be simultaneously supported.

GDF provides a superset of features from many other data formats. GDF v2.10 includes support for, user-specified event description (like in EDF+ and BrainVision format), manufacturer information (like in SCP [13] and MFER [14]), and the orientation of MEG sensors. Accordingly, GDF v2.x is (upwards) compatible with most other data formats; this means that biosignal data from other formats can be converted to GDF without loss of information. Routines for reading and writing GDF files in Octave and Matlab, as well as in C, are implemented in the open source package BioSig. For more details, please refer to [1].

## 4. BioSig in Biomedical Research

### 4.1. Heart Rate Extraction

Even the apparently simple task of extracting the heart rate from an electrocardiographic (ECG) signal is appropriate for a biosignal toolbox because the user is not distracted by coding a heart rate extractor on the fly. In BioSig, two well-tested and published algorithms [15, 16] are implemented in a single routine. The first algorithm determines the envelope of the ECG using a Hilbert transform and the positions of the R-peaks are determined by thresholding. The second algorithm uses a bank of filters, and it incorporates an ectopic beat correction. The resulting heart rates are therefore suitable for advanced heart rate variability studies.

**Figure 3:**
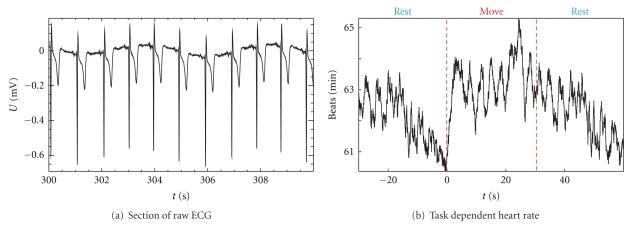
(a) Section of 10 s of raw ECG from a measurement lasting 1800 s. (b) The heart rate determined from the ECG was averaged with respect to the onset of the finger movement task performed by the subject. The changes in the averaged heart rate are within a range of 4 beats/min. This is small in relation to the 63 beats/min mean value and it can be concluded that the subject was relaxed.

The first algorithm [15] was used in the example shown in Figure 3. During a session lasting 1800 s, the MEG, the fNIRS (functional near-infrared spectroscopy), and the ECG were recorded from a subject. For the whole duration the subject had to alternate between 30 s of finger movements and 30 s of rest. The heart rate was extracted offline from the ECG. Subsequently, event-related averages were calculated for MEG, fNIRS, and heart rate over the 30 epochs of finger movements using trigger points related to the onset of finger movements. The extraction of heart rate, determination of trigger time points, and the averaging were performed using appropriate routines from BioSig. The acquisition of the ECG followed by the extraction of an event-related heart rate, as shown in Figure 3, established that the subject was in a relaxed state throughout the measurement. The oscillations in the averaged heart rate in Figure 3(b) indicate a certain degree of synchrony between respiration and the finger movement task. Results for MEG and fNIRS are discussed in [17].

### 4.2. Artifact Processing

 Several artifact processing methods are included in BioSig. The performance of the methods has been demonstrated in research and published in several papers. In the following we briefly explain some of them.

**Figure 4:**
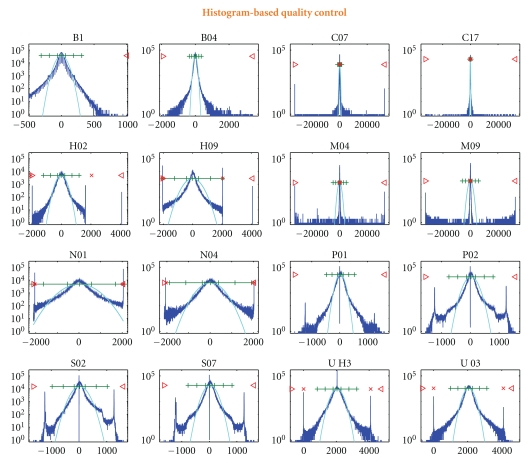
Histograms of 16 all-night sleep EEG (modified from [18]). Thresholds for overflow detection can be obtained through BioSig's eeg2hist.m tool.

The first method consists of a “histogram-based” quality control of the biomedical signals (see Figure 4). In [18] it was found that the header information of the EEG recordings does not always provide the real saturation values of the recording equipment, therefore an automated saturation detection was not possible. A quality control method based on histogram analysis was developed and its performance was successfully demonstrated. The amplitude histograms and entropies of all-night sleep recordings from 8 different sleep laboratories were calculated. This method is provided by BioSig in order to support the visual identification of the thresholds needed for the saturation detection.

Also, algorithms for detection of muscle artifacts are implemented. For example, the method described in [19] is available in BioSig. In that paper the authors used time domain and frequency domain methods for the detection of muscular noise in awake EEG. For time domain detection, they used slope and maximum/minimum amplitude. The parameters in the frequency domain were absolute and relative “high beta” power (>25 Hz) and spectral edge frequency. The detection thresholds were calculated from subject distributions calculated from a reference period. This method consistently outperforms the use of constant empirical thresholds.

Finally, a method for artifact removal of electrooculographic (EOG) artifacts in EEG is also available in BioSig. It is a very powerful algorithm based on simple linear regression. Its suitability has been demonstrated in two papers, [20, 21].

The observed EEG can be considered as a linear superposition of EEG and EOG components. This can be written in the form of a regression model:

(1) $$\begin{array}{c} {\vec{Y}}_{t}={\vec{E}}_{t}+b_{N\times M}\cdot {\vec{O}}_{t}. \end{array}$$

Accordingly, the observed EEG at time *t* is a vector ${\vec{Y}}_{t}$ with *N* elements, and the observed EOG activity ${\vec{O}}_{t}$ at time *t* has *M* elements. The observed EEG data ${\vec{Y}}_{t}$ consists of a linear superposition of the true EEG activity ${\vec{E}}_{t}$ and the ocular activity ${\vec{O}}_{t}$ that propagates through the mechanism of volume conduction to each EEG electrode. The propagation factors are described by the model parameters **b**_*N*×*M*_, which describe the influence of *M* components of the ocular dipoles to each of the *N* EEG channel. Because the propagation mechanism is simple volume conduction [22–24], it depends only on the geometry and the conductivity of the head tissue. It is reasonable to assume that these are constant during the whole EEG recording time *T*, 0 < *t* ≤ *T* and independent of frequency. It should be noted that the regression model can be also written in the following form:

(2) $$\begin{array}{c} \left[\begin{array}{c} {\vec{Y}}_{t}\\ {\vec{Z}}_{t} \end{array}\right]=\left[\begin{array}{cc} I_{N\times N} & b_{N\times M}\\ 0_{M\times N} & I_{M\times M} \end{array}\right]\cdot \left[\begin{array}{c} {\vec{E}}_{t}\\ {\vec{O}}_{t} \end{array}\right], \end{array}$$

where ${\vec{Z}}_{t}={\vec{O}}_{t}$ represents the observed EOG channels.

If the EOG activity is measured, its contribution can be removed using the least squares solution of (1). A right multiplication of (1) with ${\vec{O}}_{t}^{T}$ and applying the expectation operator 〈·_*t*_〉 over time *t* yields

(3) $$\begin{array}{c} \langle {\vec{Y}}_{t}\cdot {\vec{O}}_{t}^{T}\rangle =\langle {\vec{E}}_{t}\cdot {\vec{O}}_{t}^{T}\rangle +b_{N\times M}\cdot \langle {\vec{O}}_{t}\cdot {\vec{O}}_{t}^{T}\rangle . \end{array}$$

Because EEG and EOG can be considered uncorrelated, the term $\langle {\vec{E}}_{t}\cdot {\vec{O}}_{t}^{T}\rangle$ becomes zero, the true model coefficients are

(4) $$\begin{array}{c} b=\langle {\vec{Y}}_{t}\cdot {\vec{O}}_{t}^{T}\rangle \cdot {\langle {\vec{O}}_{t}\cdot {\vec{O}}_{t}^{T}\rangle }^{-1} \end{array}$$

and the EEG data can be corrected according to

(5) $$\begin{array}{c} {\vec{E}}_{t}={\vec{Y}}_{t}-b\cdot {\vec{O}}_{t}. \end{array}$$

The method is also known as “least squares approach” or “multiple least squares approach” in case that more than one EOG component is removed. **b** is chosen in such a way that the mean square of $\vec{E}$ is minimized.

This model takes into account only EEG and EOG sources. In practice, other noise sources (e.g., amplifier and impedance noise, electric and magnetic interferences, and muscle activity) occur, too. In order to analyze the possible influence of these noise sources on the reduction method, a noisy model has to be considered. One consequence of this analysis is the fact that the model estimates $\hat{b}=\beta$ are least biased if the signal-to-noise ratio between EOG and other noise sources is as large as possible. Therefore, we estimated the model coefficients from data with large ocular activity. Furthermore, it can be advantageous to filter the data (e.g., for removing the very low frequency components of the 1/*f* amplifier noise, and the very high frequency activity). In other words, the correction coefficients are most accurate if the influence of other noise sources can be avoided.

The difference between the regression approach (linear superposition model) and the component-based approaches such as blind source separation remains in the manner how the signals describing the EOG activity are obtained. While the regression approach uses the observed EOG activity, the component-based approaches decompose the data into a number of independent (and uncorrelated) components, and different heuristics are used for identifying the EOG components. An advantage of component-based methods could not be demonstrated within a study [20]. It is important to use bipolar EOG channels with EOG electrodes located close to the eyes as regressors. The results suggest that it is more difficult to identify the artifact components with blind source separation methods than with the dedicated channels (like EOG) recording the artifact.

**Figure 5:**
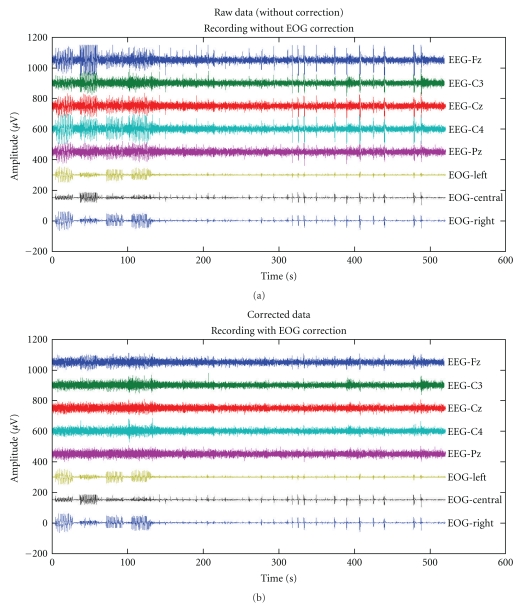
(a) Raw EEG data, contaminated with ocular artifacts. (b) Corrected data using regression analysis.

Figure 5 illustrates the regression method to correct EOG artifacts in EEG. On the left the raw EEG data is visible, on the right the corrected EEG is displayed. More detailed information is available in [20, 21].

### 4.3. Coupling and Connectivity with EEG and Multivariate Autoregressive Models

One of the most striking problems in neuroscience is the study of brain areas that interact with each other, and how they interact during the performance of a certain task.

The (auto-)spectrum of a single channel and the cross-spectrum of two different channels have been used for a while to analyze the connectivity of different brain areas [25, 26].

An often used measure related to the cross-spectrum is the coherence, which is defined as the power of the cross-spectrum of two channels normalized with the corresponding power autospectra. Therefore, its magnitude varies from 0 to +1. The normalized cross-spectrum before taking the power is called coherency, and it is a complex number, so it has a real and imaginary part. As a complex number it can be represented by its amplitude and phase.

Nolte et al. [27] proposed to investigate the imaginary part of the coherency as a connectivity measure, because a nonzero imaginary part of coherency can not be explained by volume conduction alone, but is an indicator for a functional coupling between different brain areas. By computing the phase of the coherency (using the real and imaginary parts), the time delay between signals present in two channels can be estimated. Another measure defined to remove bias due to volume conduction is the partial coherence. It is computed between a pair of channels, partializing out the activity of the remaining channels.

The measures mentioned so far are antisymmetric or symmetric and therefore cannot represent the direction of the information flow. Kaminisky and Blinowska [28] proposed the directed transfer function to detect whether the coupling between brain areas is forwards, backwards, or both. The partial directed coherence (PDC), motivated by the partial coherence, was also defined for this purpose in [29]. Only the PDC and the generalized PDC have the potential to identify the causal relationships and the underlying structure of an observed system.

All these measures have in common that they can be estimated from a multivariate autoregressive (MVAR) model, so that MVAR models can be considered a common basis for the comparison of different coupling measures. BioSig does integrate a complete toolbox for MVAR modeling in the folder * tsa* (time series analysis) [2].

Examples for the application of coupling measures to EEG using BioSig can be found in [30–33], and methodological issues are addressed in more detail by the works [8, 34].

### 4.4. Brain Computer Interfacing

 The purpose of a BCI system is to identify the user's intention by observing and analyzing brain activity without relying on signals from muscles or peripheral nerves. BioSig contains many useful tools for BCI research, most of them designed for EEG signals (although certain functions can be used to process other signals). The reason why BioSig is focused on EEG is that it is noninvasive, portable, can be used in almost any environment, and it has excellent time resolution.

**Figure 6:**
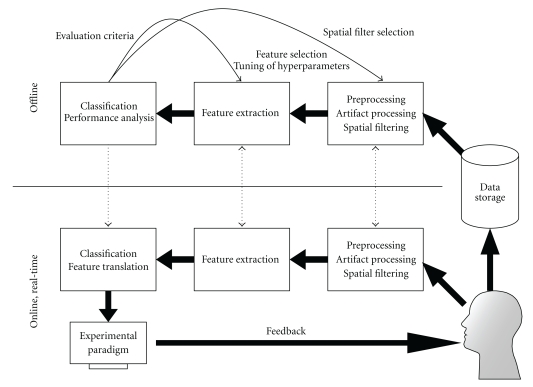
Elements of a brain computer interface.

Figure 6 illustrates a typical BCI. An online data-processing system controls devices in real time and provides feedback to the user. To generate the control signal, the BCI must extract and classify EEG features. The feature extraction method is typically based on the type of neurophysiological activation, and the classifier is usually obtained by offline analyses of previous data records from the same subject (subject-selected feature parameters as well, cf. [35]).

The rtsBCI toolbox is a real-time Brain Computer Interface (BCI) system implemented in Matlab and Simulink that can serve the purpose of designing an online system, however it is currently not supported.

Some BCIs use primarily spectral analysis (e.g., frequency band power or autoregressive spectra) to characterize spontaneous oscillatory EEG activity. It can also use autoregressive parameters directly to describe the entire spectral density function [36]. Alternatively, a BCI can analyze the user's response to visual or acoustic stimuli, which can be presented one by one or in a steady-state (repetitive) mode. 

As already mentioned in Section 4.2, data preprocessing is important to remove the influence of technical artifacts and nonbrain activity such as electrical signals caused by eye movements or facial muscles. Section 4.2 offers examples of methods that are implemented in BioSig to remove artifacts. Additionally, in the case of EEG recordings, spatial filters can also focus on a specific brain area or identify particular signal components [35, 37–39]. These are available in BioSig as well.

A BCI uses offline analysis for several purposes. The most common is the estimation of a reliable classifier. But when the classifier and/or features contain hyperparameters, such as adaptation speed or regularization coefficients, they need to be tuned off-line [40–43]. For the evaluation of methods, the application of cross-validation and may be resampling procedures might be necessary. All tools for offline analysis are as well available in BioSig.

An overview of general methods for BCI research implemented in BioSig is presented in Table 1.

**Evaluation Criteria**

Traditionally, BCI performance is quantified by classification accuracy or error rate. For the multiclass (when the user performs more than 2 tasks), there are other metrics that might be preferable as for example, the Cohen's kappa coefficient, which is derived from the confusion matrix [44].

In some cases, it is desirable to quantify BCI performance in terms of the information transfer rate [45]. Other metrics are the correlation coefficient, mean square error, and area under the receiver operating characteristic curve. Support for these and some more criteria is provided within BioSig [46–48].

## 5. BioSig for C/C++ and Libbiosig

BioSig for C/C++ (short biosig4c++) provides some command line tools for data conversion, a library to access a number of data formats (libbiosig), and some experimental code for network transfer of biosignal data. The motivation for a C/C++-based library is performance issues, flexibility in terms of supported platforms (e.g., Matlab can be hardly installed on some embedded devices), and interfacing to existing libraries. When there was a need for a converter between XML-based HL7aECG and the SCP-ECG data format, it was first implemented in C/C++ [49], nowadays about 30 data formats can be read and 10 are written. Moreover, biosig4c++ can be used now with Octave and Matlab through a MEX-interface yielding a much better performance than the traditional m-scripts. biosig4c++ provides also an interface for Python (it enables reading of 30 biosignal data formats into Python) and might be also useful for other software platforms. The free viewing and scoring software “SigViewer” uses libbiosig for accessing biosignal data. An experimental implementation of a network-based data transfer is included in biosig4c++, which might be useful for biosignal recorders (embedded devices) and for network-based biosignal archives.

## 6. Conclusions and Future Work

 BioSig provides a whole tool chain of data processing methods for BCI research. These tools are useful in other application areas, like seizure detection and seizure prediction in epileptic EEG. Connectivity analysis with MVAR methods is another expanding application topic of BioSig.

Data analysis with biosig4octmat emphasizes the almost fully automated data analysis. However, a major limitation to this goal is the manual scoring of artifacts. Here, we see a need to validate promising artifact processing methods. The results on EOG artifacts show that a two-channel regression analysis can reduce about 80% of EOG artifacts in EEG recordings [21], similarly to blind source separation methods combined with some heuristics for component selection, [20]. Similar results are expected for raw MEG data in channel space. For EMG artifacts, there are methods of inverse filtering [36] and high pass filtering implemented [50], but currently only limited results about their performance are available. However, the validation of the performance of artifact processing methods is crucial for a number of possible applications, including BCI but also seizure detection and prediction.

Up to now, controlled signal conditioning under BioSig is not included in order to simplify the design and also because during the experiments the conditions are usually fixed. Otherwise it is not clear whether changes are due to the recording system or they occurred in the observed system.

biosig4octmat is an application that can be used with Octave and is available from Sourceforge. Currently, the monthly download range is about 500 per month. Its installation in Octave is similar as in Matlab. However, the BioSig benchmark shows that Octave is somewhat slower than Matlab. In the future, the BioSig development will stay committed to compatibility to Octave, and will work actively to support compatibility with both. 

For certain applications, the support of hardware platforms beyond personal computers is of interest. For example, embedded devices are important for online and real-time applications. Here, a C/C++ library like biosig4c++/libbiosig can be very useful. Biosig supports so far different programming languages including C/C++, Octave/Matlab and Python. Experimental support for other programming languages (e.g., Java, PhP, Perl, Ruby, Tcl, etc.) using the SWIG tool is currently investigated. Standardization (of data formats as well as data analysis methods) is also an important area, and due to its free software development model BioSig provides a suitable platform for these topics. 

So far, the emphasis of BioSig was in providing a library of high-quality methods, useful algorithms, and reference implementations for all kind of biomedical signal processing problems, rather than a “user-friendly” environment for nonexperts. However, a folder with several Demos is available after installation (biosig/demo/). The future of BioSig is open, and the development and future direction of BioSig depends on each contributor to the BioSig project.

## Figures and Tables

**Table 1:** List of BCI-related task that can be performed using BioSig.

Data preprocessing	Triggering, partitioning of data
	Artifact processing
	Quality check of data through histogram analysis
	Spatial filters
	Detection of EMG artifacts
	Common spatial patterns

Feature extraction Band power	Adaptive autoregressive parameters
	Adaptive multivariate autoregressive parameters
	(Adaptive) Hjorth
	(Adaptive) Barlow
	(Adaptive) Wackermann
	(Adaptive) time-domain parameters
	Adaptive brain rate,
	spectral edge frequency

Feature classification	Linear discriminant analysis (LDA)
	Quadratic discriminant analysis (QDA)
	Support vector machines
	Naive Bayesian classifier (NBC)
	Augmented NBC
	Sparse LDA
	Generalized discriminant analysis

Evaluation criteria Classification accuracy	Cohen's kappa coefficient
	Receiver operating characteristics (ROC)
	Area under the ROC curve
	Mutual information, information transfer rate
	Correlation coefficient

Metafunctions	findclassifier, cross-validation (xval),
	standardized analysis
	(demo2 is an example of a standardized
	offline analysis)
